# Supplementary material for: Sorting at embryonic boundaries requires high heterotypic interfacial tension
Source: Nat Commun. 2017 Jul 31;8:157. doi: 10.1038/s41467-017-00146-x (PMC5537356; doi:10.1038/s41467-017-00146-x)
Supplement: Supplementary file 2 — Supplementary Software 1 [file 41467_2017_146_MOESM2_ESM.zip › PottsModel/SrcPottsModel/doc/model/class-use/Cell.CellType.html]

Uses of Class model.Cell.CellType


JavaScript is disabled on your browser.


Skip navigation links


- Overview
- Package
- Class
- Use
- Tree
- Deprecated
- Index
- Help

- Prev
- Next

- Frames
- No Frames

- All Classes

## Uses of Class model.Cell.CellType

- Packages that use Cell.CellType

  | Package | Description |
  | --- | --- |
  |  |  |
  | --- | --- |
  | engine |  |
  | model |  |
- - ### Uses of Cell.CellType in engine

    Constructors in engine with parameters of type Cell.CellType

    Constructor and Description

    `DispersionIndex(PottsEngine engine,
    Cell.CellType[] pCellTypes)`

    `DispersionIndex(PottsEngine engine,
    Cell.CellType[] pCellTypes,
    int frequency)`

    `HMDStatistic(PottsEngine pEngine,
    Cell.CellType[] pCellTypes,
    int pFrequency)`

    `IsoperimetricQuotientStatistic(PottsEngine pEngine,
    Cell.CellType[] pCellTypes,
    int pFrequency)`

    `TypeSpecificAreaStatistic(PottsEngine pEngine,
    Cell.CellType[] pCellTypes,
    int pFrequency)`

    `TypeSpecificCellStatistic(PottsEngine pEngine,
    java.lang.String pYAxisLabel,
    Cell.CellType[] pCellTypes,
    int pFrequency)`

    `TypeSpecificNearestNeighborStatistic(PottsEngine pEngine,
    Cell.CellType[] pCellTypes,
    int pFrequency)`

    `TypeSpecificNearestNeighborStatistic(PottsEngine pEngine,
    Cell.CellType[] pCellTypes,
    int pFrequency,
    int pNumNeighbors)`

    `TypeSpecificNumNeighborsStatistic(PottsEngine pEngine,
    java.lang.String pYAxisLabel,
    Cell.CellType[] pCellTypes,
    int pFrequency)`

    `TypeSpecificPercentIsolatedCellStatistic(PottsEngine pEngine,
    java.lang.String pYAxisLabel,
    Cell.CellType[] pCellTypes,
    int pFrequency)`

    `TypeSpecificPerimeterStatistic(PottsEngine pEngine,
    Cell.CellType[] pCellTypes,
    int pFrequency)`

    `TypeSpecificStatistic(PottsEngine pEngine,
    java.lang.String pYAxisLabel,
    Cell.CellType[] pCellTypes,
    int pFrequency)`
  - ### Uses of Cell.CellType in model

    Methods in model that return Cell.CellType

    | Modifier and Type | Method and Description |
    | --- | --- |
    |  |  |
    | --- | --- |
    | `Cell.CellType` | Pixel.`getCellType()` |
    | `static Cell.CellType` | Cell.`getCellTypeFromID(int pID)` |
    | `Cell.CellType` | Cell.`getType()` |
    | `static Cell.CellType` | LatticeUtilities.`inverse(Cell.CellType c)` |
    | `static Cell.CellType` | Cell.CellType.`valueOf(java.lang.String name)` Returns the enum constant of this type with the specified name. |
    | `static Cell.CellType[]` | Cell.CellType.`values()` Returns an array containing the constants of this enum type, in the order they are declared. |

    Methods in model with parameters of type Cell.CellType

    | Modifier and Type | Method and Description |
    | --- | --- |
    |  |  |
    | --- | --- |
    | `static java.awt.Color` | Cell.`getCellTypeColor(Cell.CellType pType)` |
    | `static java.lang.String` | Cell.`getCellTypeColorName(Cell.CellType pType)` |
    | `double` | Constants.ParameterManager.`getCellTypeInteractionEnergy(Cell.CellType t1, Cell.CellType t2)` |
    | `static Pixel.PixelType` | Pixel.`getDefaultPixelType(Cell.CellType pCellType)` |
    | `static Cell.CellType` | LatticeUtilities.`inverse(Cell.CellType c)` |
    | `void` | Constants.ParameterManager.`setInteractionEnergy(Cell.CellType t1, Cell.CellType t2, double n)` |

    Method parameters in model with type arguments of type Cell.CellType

    | Modifier and Type | Method and Description |
    | --- | --- |
    |  |  |
    | --- | --- |
    | `void` | Constants.ParameterManager.`setCellTypeInteractionEnergies(java.util.HashMap<java.util.EnumSet<Cell.CellType>,java.lang.Double> cellTypeInteractionEnergies)` |

    Constructors in model with parameters of type Cell.CellType

    Constructor and Description

    `Cell(Cell.CellType pType,
    int pID,
    Lattice pLattice)`

    `SquareCell(Cell.CellType pType,
    int pID,
    Lattice pLattice)`

Skip navigation links


- Overview
- Package
- Class
- Use
- Tree
- Deprecated
- Index
- Help

- Prev
- Next

- Frames
- No Frames

- All Classes
